# Difficult Airway Management Algorithm in Emergency Medicine: Do Not Struggle against the Patient, Just Skip to Next Step

**DOI:** 10.1155/2010/826231

**Published:** 2010-07-27

**Authors:** Jérôme Sudrial, Caroline Birlouez, Anne-Laurette Guillerm, Jean-Luc Sebbah, Roland Amathieu, Gilles Dhonneur

**Affiliations:** ^1^Prehospital Emergency Medicine Department, General Hospital Gonesse, 95500, France; ^2^Anesthesia and Intensive Care and Emergency Medicine Department, Jean Verdier University Hospital of Paris, 93143 Bondy, France; ^3^Département d'Anesthésie et Réanimation, CHU de l'APHP, Jean Verdier, Av du 14 Juillet, 93143 Bondy, France

## Abstract

We report a case of prehospital “cannot intubate” and “cannot ventilate” scenarios successfully managed by strictly following a difficult airway management algorithm. Five airway devices were used: the Macintosh laryngoscope, the gum elastic Eschmann bougie, the LMA Fastrach, the Melker cricothyrotomy cannula, and the flexible fiberscope. Although several airway devices were used, overall airway management duration was relatively short, at 20 min, because for each scenario, failed primary and secondary backup devices were quickly abandoned after 2 failed attempts, each attempt of no more than 2 min in duration, in favor of the tertiary rescue device. Equally, all three of these rescue devices failed, an uncuffed cricothyroidotomy cannula was inserted to restore optimal arterial oxygenation until a definitive airway was secured in the ICU using a flexible fiberscope. Our case reinforces the need to strictly follow a difficult airway management algorithm that employs a limited number of effective devices and techniques, and highlights the imperative for early activation of successive preplanned steps of the algorithm.

## 1. Introduction

We report a case of difficult airway management performed in the prehospital environment. Initial attempts at airway management revealed a “cannot intubate” scenario followed by a “cannot ventilate” scenario. Although primary and secondary backup devices failed, tertiary rescue devices succeeded for each arm of the algorithm. Our case reinforces the imperative for strictly following a difficult airway management algorithm (DAMA) that includes several airway devices; that the algorithm should be rapidly navigated is necessary as well as activation of successive preplanned steps of the algorithm.

## 2. Case Report

The medical emergency medical team of the prehospital unit of a general hospital was sent to the house of a 57-year-old morbidly obese patient (BMI = 40 kg·m^−2^) because of status epilepticus. 

Upon arrival on the scene, the patient was found lying on the floor. Initial vital signs were as follows: pulse 115  min^−1^, respiration 21  min^−1^, blood pressure (S/D) 175/105 mmHg, and arterial oxygen saturation 83%. The Glasgow Coma Scale was 7 (E = 2 − V = 2 − M = 3). Chest auscultation revealed that gastric content aspiration had probably occurred. Tracheal intubation was urgently indicated. After a short period of bag-mask oxygenation, blow by oxygen (12 L·min^−1^), arterial oxygenation with spontaneous ventilation reached 93%–94%. Sellick maneuver was applied prior to performing a rapid sequence induction using thiopental (300 mg) and succinylcholine (100 mg). 

The first laryngoscopy performed with a single-use metallic Macintosh blade (size 3) revealed a Cormack and Lehane grade 3 view, that was unmodified when Sellick's maneuver was released and external laryngeal manipulations (including BURP maneuver) performed. After the first failed tracheal intubation attempt, the Macintosh blade was changed for a size 4, followed by a second failed attempt. A gum elastic Eschmann bougie was employed while direct laryngoscopy was maintained in an attempt to blindly intubate the trachea. Unfortunately, the bougie was routed into the esophagus (no hold-up sensation) on two occasions. A four-handed attempt at facemask ventilation assisted with Guedel oropharyngeal airway failed to improve oxygenation with arterial oxygen saturation as low as 75% SpO2.

The treating physician then decided to use the LMA Fastrach (SEBAC, Pantin, France) after a second bolus of succinylcholine (100) mg and thiopental (200 mg) was injected. Just after insertion and inflation of the cuff of a size 4 reusable LMA Fastrach, the Chandy maneuver allowed some ventilation to be established, increasing the SpO2 to 96%. The first blind tracheal intubation attempt through the LMA Fastrach resulted in a probable esophageal intubation as suggested by capnography and abdominal auscultation. During the second tracheal intubation attempt performed with a slow maneuver which the physician applied to the laryngeal mask, the physician was unable to push the endotracheal tube out of the laryngeal mask's metallic tube. 

Unfortunately, bag ventilation through the LMA Fastrach became more difficult and arterial oxygenation deteriorated to 72%. For this reason a cricothyroidotomy un-cuffed cannula (Melker, COOK, Charenton-le-Pont, France) was rapidly inserted in the trachea (<60 seconds). All the while, the LMA Fastrach was kept in place and bag ventilation attempts continued. After suctioning of tracheal and bronchial secretions and bag ventilation through the cricothyroidotomy cannula, SpO2 improved. 

Interestingly, a gas leak sound perceived at the proximal end of the metallic tube of the LMA Fastrach was noted to occur with bag ventilation insufflations. Digital occlusion of the proximal end of the LMA Fastrach dramatically improved bag ventilation quality and promoted clear breath sounds on chest auscultation associated with 100% SpO2. 

After approximately 20 minutes of difficult airway management activity, the patient's airway was secure and the patient was transported to the district hospital for cerebral scanning. Metal-related artifact on CT due to the metallic reusable LMA Fastrach prompted its removal which was not associated with change in the oxygenation quality. A nasogastric tube was placed to continuously suction the stomach contents. Upon arrival in the triage area, an emergency medicine physician fiberoptically assisted nasotracheal intubation. The patient was extubated on day 4 and discharged from the ICU on day 7.

## 3. Discussion

**Figure 1:**
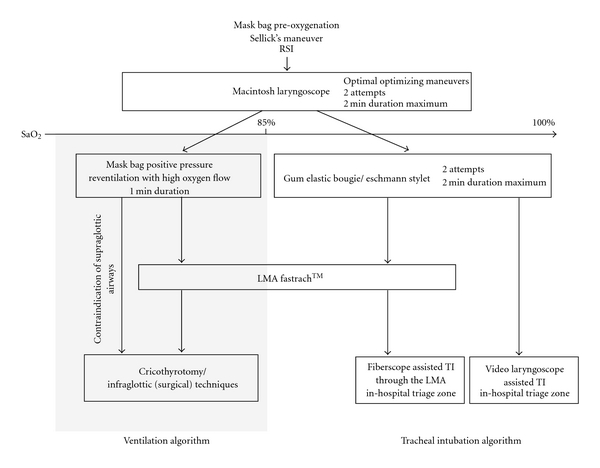
Difficult Airway Management Algorithm.

We report a case of difficult prehospital airway management beginning with an impossible intubation scenario followed by an impossible ventilation scenario. The senior emergency medicine physician on scene successfully managed this difficult case by strictly following a DAMA. For each arm (i.e., cannot intubate and cannot ventilate) of the DAMA (Figure 1), the physician followed evidence-based clinical [1, 2] and expert panel's [3, 4] recommendations by using step-by-step approach to secure the airway and maintain oxygenation.

Unfortunately, at each arm of the algorithm, primary and secondary airway devices failed, whereas the tertiary backup strategies succeeded. An uncuffed cricothyroidotomy cannula favored the reestablishment of optimal arterial saturations, permitting leisurely approach to definitive airway management employing a flexible fiberscope and nasotracheal technique.

Our observation confirms that when conventional airway management methods fail, accessory rescue airway devices rapidly deployed in a predefined algorithmic fashion assist the clinician in managing the airway. Clinicians cannot rely on a single airway device, but rather on several devices because each of them has an intrinsic failure rate [5]. In the present paper, five airway devices were used: the Macintosh laryngoscope, the gum elastic Eschmann bougie, the LMA Fastrach, the Melker cricothyroidotomy cannula, and the flexible fiberscope. Although several airway devices were used, overall airway management duration was relatively short, because for each scenario, failed primary and secondary backup devices were quickly abandoned after 2 failed attempts, with 2 minutes duration limit for each attempt, to rapidly use the tertiary rescue device.

Our observations reinforce the need for predefined DAMA to safely manage difficult airway cases. These DAMAs should include a variety of validated rescue devices. All physicians must be trained with each of them. Once these DAMAs are accepted and the physicians are skilled in multiple airway tools, difficult airway management simulation programs should focus upon the following:

1. a precise, strict, and acceptable definition of device-related failure,
2. proposing an acceptable 2-minute duration limit of each airway device attempt, and
3. setting lower limits for a relevant physiological parameter such as SpO2 (e.g., <85 %) for the activation of a rescue infraglottic (surgical) oxygenation procedure.

Interestingly, the LMA Fastrach succeeded in establishing effective ventilation and oxygenation, but eventually failed to maintain a patent airway. Failure to intubate with the LMA Fastrach, has already been described [5], even in expert's hands such as those of the senior physician who managed this patient. This physician is considered to be a difficult airway expert and provides training for the juniors, residents, and staff of our department. He is also one of the participants involved in the management of the simulation-training center we have created in our university teaching hospital.

The irreversible secondary loss of the airway with the LMA Fastrach has never been described. The reasons for this persistent secondary failed ventilation despite repositioning maneuvers are presumably due to a down-folded epiglottis that probably covered the glottis and obstructed the airway. The fact that the LMA Fastrach failed in being an insufflation tool, but remained an “exsufflation” device during bag ventilation through the cricothyroidotomy un-cuffed cannula reinforces this hypothesis. 

The use of new airway devices using video-assisted laryngoscopy might have been interesting in the present case. By using a video laryngoscope, orotracheal intubation performed in the triage area under suitable conditions would have advantageously replaced, fiberscope assisted nasotracheal intubation. Indeed, the new devices equipped with viewing systems have almost systematically demonstrated their tracheal intubation efficiency in case of Macintosh laryngoscope failure despite gum elastic bougie use. 

In conclusion, our paper confirms the rationale for strictly applying a DAMA that includes several backup airway devices, each of them having failure, unambiguously defined and accepted. Struggling with the patient's airway is known to lead to a poor outcome. It is now time to identify that the early performance of infraglottic (surgical) airway techniques may be life saving.

## References

[1] Combes X, Le Roux B, Suen P (2004). Unanticipated difficult airway in anesthetized patients: prospective validation of a management algorithm. *Anesthesiology*.

[2] Combes X, Jabre P, Jbeili C (2006). Prehospital standardization of medical airway management: incidence and risk factors of difficult airway. *Academic Emergency Medicine*.

[3] Caplan RA, Benumof JL, Berry FA (2003). Practice guidelines for management of the difficult airway: an updated report by the American Society of Anesthesiologists Task Force on Management of the Difficult Airway. *Anesthesiology*.

[4] Cros A-M (2008). The consensus conference on difficult airway management. *Annales Francaises d’Anesthesie et de Reanimation*.

[5] Mort TC (2006). Laryngeal mask airway and bougie intubation failures: the combitube as a secondary rescue device for in-hospital emergency airway management. *Anesthesia and Analgesia*.

